# Immune targeting of autocrine IGF2 hampers rhabdomyosarcoma growth and metastasis

**DOI:** 10.1186/s12885-019-5339-4

**Published:** 2019-02-07

**Authors:** Carla De Giovanni, Patrizia Nanni, Lorena Landuzzi, Marianna L. Ianzano, Giordano Nicoletti, Stefania Croci, Arianna Palladini, Pier-Luigi Lollini

**Affiliations:** 1 Laboratory of Immunology and Biology of Metastasis, Department of Experimental, Diagnostic and Specialty Medicine, University of Bologna, Viale Filopanti 22, I-40126 Bologna, Italy; 2 Laboratory of Experimental Oncology, IRCCS Istituto Ortopedico Rizzoli, Bologna, Italy; 3 Present address: Unit of Clinical Immunology, Allergy and Advanced Biotechnologies, Azienda Unità Sanitaria Locale-IRCCS, Reggio Emilia, Italy

**Keywords:** IGF2, Immunoprevention, Rhabdomyosarcoma, IGF1R, DNA vaccines, Neutralizing antibodies

## Abstract

**Background:**

Insulin-like Growth Factor Receptor-1 (IGF1R) system sustains the genesis of rhabdomyosarcoma through IGF2 autocrine overexpression. While several IGF1R-targeted strategies have been investigated to interphere with rhabdomyosarcoma growth, no attempt to neutralize IGF2 has been reported. We therefore studied the possibility to hamper rhabdomyosarcoma growth with passive and active immune approaches targeting IGF2.

**Methods:**

A murine model developing IGF2-overexpressing pelvic rhabdomyosarcoma, along with IGF2-independent salivary carcinoma, was used to investigate the efficacy and specificity of passive anti-IGFs antibody treatment. Active vaccinations with electroporated DNA plasmids encoding murine or human IGF2 were performed to elicit autochthonous anti-IGF2 antibodies. Vaccinated mice received the intravenous injection of rhabdomyosarcoma cells to study the effects of anti-IGF2 antibodies against developing metastases.

**Results:**

Passive administration of antibodies neutralizing IGFs delayed the onset of IGF2-overexpressing rhabdomyosarcoma but not of IGF2-independent salivary carcinoma. A DNA vaccine against murine IGF2 did not elicit antibodies, even when combined with Treg-depletion, while a DNA vaccine encoding the human IGF2 gene elicited antibodies crossreacting with murine IGF2. Mice with anti-IGF2 antibodies were partially protected against the metastatic growth of IGF2-addicted rhabdomyosarcoma cells.

**Conclusions:**

Immune targeting of autocrine IGF2 inhibited rhabdomyosarcoma genesis and metastatic growth.

## Background

Rhabdomyosarcoma is characterized by the overexpression of Insulin-like Growth Factor-2 (IGF2) that sustains growth in an autocrine way, interacting with the Insulin-like Growth Factor Receptor-1 (IGF1R) [1–4]. IGF2 overexpression can be caused by a loss of imprinting or heterozygosity at the 11p15.5 locus and plays a role in rhabdomyosarcoma pathogenesis [1–5]. IGF2-targeted siRNA cause a decreased in vitro growth of rhabdomyosarcoma [6]. Since IGF2 is involved both in etiogenesis and in growth of rhabdomyosarcoma, the interruption of this autocrine circuit could have both preventive and therapeutic effects.

Several therapeutic strategies targeting IGF1R are reported in the literature [7]. Passive administration of anti-IGF1R antibodies can block the autocrine system, thus inhibiting rhabdomyosarcoma tumor growth [8, 9]. Anti-IGF1R passive approaches based on IGF1R-neutralizing monoclonal antibodies were studied in therapeutic clinical trials [10, 11]. Treatment was safe but had limited activity [10, 11]. An attempt to obtain an active immune response against IGF1R has been reported in a mammary cancer model [12].

The interruption of IGFs autocrine rhabdomyosarcoma circuits targeting IGF2 through neutralizing antibodies was not studied so far. Immune targeting of IGFs has been reported in a few non-rhabdomyosarcoma experimental models in which IGFs played a role [13–16]. Therapeutic approaches showed some effectiveness of anti-IGFs antibodies against growth of human xenograft models of prostatic cancer bone metastases [13] and of colorectal cancer metastasis [14, 16], whereas a preventive effect was exerted by anti-IGFs antibodies against development of intestinal polyps in the Apc^min^ murine model [15]. Primary prevention of rhabdomyosarcoma could have a very limited field of application, restricted to some genetic syndromes with high incidence of rhabdomyosarcoma [17]. Prevention of metastasis development after primary cancer surgery could have a major impact in rhabdomyosarcoma survival.

In this paper we investigated passive or active immune neutralization of IGF2 to interrupt IGF2-based autocrine circuits in experimental models of rhabdomyosarcoma.

## Methods

### Mice, cells and treatments

In our animal facilities BALB/c p53+/− female mice (BALB/cJ-Trp53tm1Tyj, purchased from The Jackson Laboratory, Bar Harbor, MI) were crossed with BALB/c HER2/neu transgenic male mice [18], carrying a mutant rat Neu oncogene under control of a MMTV-LTR. Mice bearing the p53^+/−^/Neu^+/−^ genotype (referred to as BALB-p53Neu) were selected by PCR genotyping. Male BALB-p53Neu mice develop salivary gland carcinomas and IGF2-overexpressing pelvic rhabdomyosarcomas in urethral tissue proximal to bladder at about 13–15 weeks of age [19]. Wild-type BALB/c AnNCrlBR (BALB/c) were purchased from Charles River Italy.

The following cell lines were used throughout the study: RMSp53Neu-5, derived from a rhabdomyosarcoma of BALB-p53Neu male mice [19]; TS/A, derived from a mammary carcinoma arisen in a BALB/c female retired breeder mouse [20]. Adherent cell cultures were grown in Dulbecco’s MEM supplemented with 10–20% Fetal Bovine Serum. For anchorage independent growth, cells were seeded at 1000 or 2000 cell/cm^2^ in 6-well Multiwell plates in culture medium supplemented with 0.33% agar (Sea-Plaque™ Agarose, Lonza) over an underlayer of 0.5% agar medium. The IGF1R kinase inhibitor NVP-AEW541 (kindly provided by Novartis Pharma, Basel, Switzerland) was added to medium at doses ranging 0.1 to 3 μM. Controls contained vehicle alone (DMSO). Colonies were counted after 17–22 days.

Vaccinated (*n* = 5) and control mice (*n* = 6) were challenged with murine rhabdomyosarcoma cells RMSp53Neu-5 administered by intravenous injection (3 × 10^5^ cells in 0.4 ml PBS). Four weeks after, mice were euthanized with inhalation of carbon dioxide, and killing was completed by cervical dislocation. Mice were subjected to an accurate necropsy, their lungs were fixed in Fekete’s solution and metastases were counted under a dissection microscope.

### Monoclonal antibodies against IGFs

Monoclonal antibodies neutralizing IGFs (kindly provided by Kyowa Hakko Kirin Co, Tokyo, Japan) were KM1468 (rat IgG2b, neutralizes human IGF1 and IGF2 and murine IGF2 but not murine IGF1 and human insulin) and KM3168 (rat IgG2a, neutralizes human and murine IGF1, but not human and murine IGF2 and insulin) [15]. BALB-p53Neu male mice at a pre-neoplastic stage (5–6 weeks of age) were randomized based on the weeks of age to three experimental groups: control and two doses of a mixture of IGFs MAbs KM1468 and KM3168 (0.2 and 1 μg/g for each antibody). Mice received two administrations per week in the site of onset of rhabdomyosarcoma for a total of 18 injections. Control group received only vehicle (phosphate-buffered saline). Mice were monitored twice weekly for tumor growth by palpation. Tumor growth was periodically monitored through measure of diameters (*a* = maximal tumor diameter and *b* = major tumor diameter perpendicular to *a*) with calipers; tumor volumes were calculated as π[√(*a* × *b*)]^3^/6 as reported in [21]. Mice were euthanized as above according to the criteria for standardized and measurable humane endpoints approved by the Institutional Animal Care and Use Committee of the University of Bologna. Time to sacrifice was assumed as overall survival.

### Plasmids, transfections and DNA vaccinations

Plasmidic pBLAST49-derived expression vectors for murine IGF2 (p-mIGF2) and human IGF2 (p-hIGF2), as well as the empty vector pBLAST49-mcs, were purchased from InvivoGen (San Diego, USA). For transient transfection of TS/A cells, 24 h after seeding cells were transfected with 1 μg of plasmid coding for mIGF2 or hIGF2 and with 2.5 μl of Lipofectamine 2000 (Thermo Fisher Scientific) according to the manufacturer protocol. Supernatants were collected after 72 h culture, and release of mIGF2 or hIGF2 was determined by ELISA using DuoSet Elisa Development system (R&D Systems, Inc., Minneapolis, USA). For IGF1R silencing two siRNA, siRNA-R1 and siRNA-R4 (Qiagen, Milan, Italy) directed against two different regions of IGF1R transcript, were used and compared to control siRNA not homologous to any mouse mRNA [22, 23]. Cells were cultured for 48 h in the presence of siRNA at 40 nM concentration using Oligofectamine (Thermo Fischer Scientific) as transfection agent (0,8%). Then cells were harvested and reseeded in medium containing 0.33% agar without siRNA over a 0.5% agar underlayer medium. Colony growth was monitored weekly and determined by counting at low magnification (25×) 14 days after seeding.

Largescale production and purification of the plasmids were performed with EndoFree Plasmid Giga kits (QIAGEN, Valencia, CA, USA). Anesthetized BALB/c mice (8–10 weeks-old) received DNA vaccination through the injection into the tibial muscles of 50 μg of plasmid diluted to a final volume of 40 μl (20 μl in each muscle through a 28-gauge needle) in final concentrations of 0.9% NaCl and 6 mg/ml polyglutamate. Immediately thereafter electroporation was performed: two square wave, 25-ms, 375 V/cm pulses were generated with a T830 electroporator (BTX, San Diego, CA, USA) [21]. Vaccination was repeated after 2, 6 and 8 weeks, for a total of 4 vaccinations. In some experiments, the first two vaccinations were preceeded by Treg depletion at day − 1, through the intraperitoneal injection of 500 μl anti-CD25 PC61 monoclonal antibody, kindly provided by Dr. Silvano Ferrini, Istituto Nazionale per la Ricerca sul Cancro, Genoa [24].

### Immune response

Mice were routinely bled via the tail vein and serum samples were stored frozen at − 80 °C. Production of anti-IGF2 antibodies was analyzed by Western Blot. One μg of recombinant mouse or human IGF2 (R&D System) was run on a 20% polyacrylamide gel. After blocking, membranes were cutted and incubated with serum of vaccinated or untreated mice diluted 1:100 in blocking buffer. Monoclonal rat anti-IGF2 antibody (clone #122404, R&D Systems, Inc., Minneapolis, USA), that shows cross-reactivity with recombinant human/mouse IGF2, was used as positive control at the concentration of 1.7 μg/ml.

A specific Enzyme-Linked Immunosorbent Assay (ELISA) to evaluate the levels of anti-IGF2 antibodies was performed as follows: Thermo Scientific Immunoplate Nunc Maxisorp 96-well microplates (Cole-Parmer North America, Vernon Hills, CA, USA) were coated with recombinant mouse or human IGF2 at 2 μg/ml in 100 μl by overnight incubation. After blocking in Plasma Sample diluent 2x (ImmunoChemistry Technologies) + PBS 0.05% Tween20 and washing incubations, sera of vaccinated or untreated mice were added at 1:100 dilution in blocking buffer. Serum samples obtained after the fourth vaccination were used. Reaction was revealed by adding secondary goat anti-mouse immunoglobulin G (IgG)-peroxidase conjugated antibody (1:10000 dilution; Calbiochem, San Diego, CA, USA) and then 3,3′,5,5′-tetramethylbenzidine peroxidase substrate (Thermo Scientific, Rockford, IL, USA). Then 0.18 M sulfuric acid was added to stop the reaction. Absorbance at 450 nm and 620 nm was determined through an ELISA microreader (Tecan Systems, San Jose, CA, USA). Mouse monoclonal anti-human IGF2 antibody, clone 75,015.11, (R&D Systems, Inc., Minneapolis, USA), which shows 100% cross-reactivity with murine IGF2, was used to set up a standard curve run in parallel (0.05 to 200 ng/ml).

### Statistical analysis

Differences in tumor-free survival curves were analyzed by the Mantel-Haenszel test. Antibody levels were compared by the Student’s t test or the nonparametric Wilcoxon test. Metastases number were compared by the nonparametric Wilcoxon test.

## Results

### Autocrine IGF2 circuit in the BALB-p53Neu murine model of rhabdomyosarcoma

**Fig. 1:**
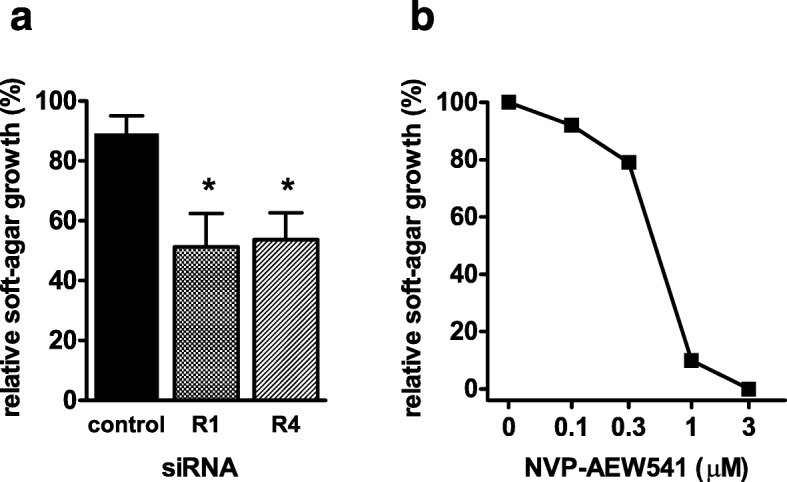
IGF1R dependence of murine rhabdomyosarcoma RMSp53Neu-5 cell line. Soft agar growth is inhibited by IGF1R-neutralizing approaches. **a** Effect on agar growth of two different siRNAs against IGF1R (R1 and R4). Control bar refers to cells cultured in the presence of control siRNA not homologous to any mouse mRNA. Percentage of growth relative to oligofectamine only is shown. Star: significantly different from control siRNA (*p* < 0.05 at Student’s *t* test). **b** Dose-related growth inhibition in the presence of the IGF1R inhibitor NVP-AEW541. Dose “0” corresponds to controls containing vehicle alone

BALB-p53Neu male mice, which carry a p53 null allele and a HER2/neu heterozygous transgene, develop pelvic rhabdomyosarcomas, at a median age of 14 weeks, along with almost concomitant salivary gland carcinomas [19]. We previously found that rhabdomyosarcomas, but not salivary gland carcinomas, overexpressed IGF2 concomitant to membrane IGF1R, thus suggesting that, like the human counterpart, experimental rhabdomyosarcoma could harbor an autocrine IGF circuit [19]. To verify the IGF2 dependence of such murine rhabdomyosarcoma model, we obtained a rhabdomyosarcoma cell line (RMSp53Neu-5) and we treated it in vitro with NVP-AEW541, a small molecule inhibitor of IGF1R, or with specific siRNAs (Fig. 1). Both treatments inhibited the 3D growth of RMSp53Neu-5 cells, thus confirming the existence of an autocrine loop acting through IGF1R.

### Prevention of rhabdomyosarcoma by passive administration of anti-IGFs antibodies

**Fig. 2:**
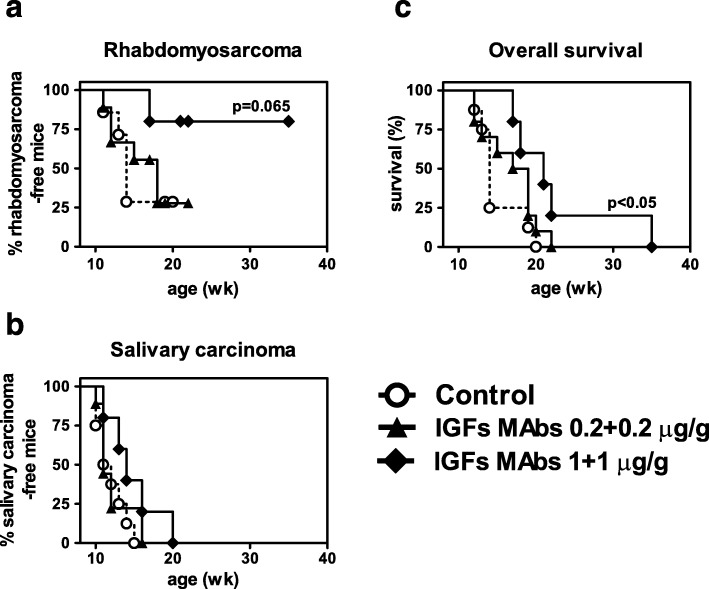
Prevention of spontaneous rhabdomyosarcoma in BALB-p53Neu male mice by passive administration at the site of rhabdomyosarcoma onset of IGFs-neutralizing Monoclonal Antibodies (IGFs MAbs). IGFs MAbs consisted of a 1:1 mixture of KM3168 + KM1468 monoclonal antibodies. **a** Rhabdomyosarcoma tumor-free survival. **b** Salivary carcinoma-free survival. **c** Overall survival (as defined in Materials and Methods). Symbols and number of mice per group: open circles: controls (vehicle alone), *n* = 7; triangles: IGFs MAbs 0.2 + 0.2 μg/g, *n* = 9; diamonds: IGFs MAbs (1.0 + 1.0 μg/g), *n* = 5. Statistical significance by the Mantel-Haenszel test versus untreated controls is reported inside each panel

To test whether immune targeting of the autocrine IGF loop might affect rhabdomyosarcoma onset, we treated young, tumor-free BALB-p53Neu male mice with antibodies against IGFs. These mice develop almost simultaneously IGF2-dependent rhabdomyosarcoma and IGF2-independent salivary carcinoma, thus allowing to evaluate the specificity of anti-IGFs treatment. Schedules and doses of antibodies were chosen as reported in non-rhabdomyosarcoma models, where pharmacokinetics data were also reported [13–15]. Passive administration of anti-IGFs antibodies caused a dose-related delay in the onset of rhabdomyosarcoma (Fig. 2a), while onset of salivary carcinoma was unaffected (Fig. 2b). The significant increase in the overall survival was likely due to the delayed rhabdomyosarcoma onset (Fig. 2c). Due to the early onset of spontaneous tumors and to the early upregulation of IGF2 in preneoplastic urethral tissue [25], BALB-p53Neu mice entered the treatment at young age (5–6 weeks) and were treated up to 14 weeks of age, therefore treatment coincided with the period of weight gain. No side effect was observed and weight gain throughout the treatment was about 22% in all the experimental groups (data not shown), according to data obtained with a non-rhabdomyosarcoma model [15].

### Induction and effectiveness of antibodies against IGF2

The induction of antibodies against mIGF2 should depend upon the breakage of tolerance against a self-molecule. We used as DNA vaccines two expression plasmids carrying murine or human IGF2 gene isoform, the latter case to take advantage of a possible adjuvant effect of the xenogeneic, even though highly homologous, molecule [26]. These vectors were able to induce good IGF2 expressions in a murine recipient cell line (Table 1). Administration of DNA vaccine was followed by electroporation, which constitutes per se an immunological adjuvant [27]. Moreover, in some experiments we combined DNA vaccine against the murine IGF2 isoform with Treg depletion.

**Table 1:** Expression vectors for IGF2 and ability to transfer IGF2 expression in TS/A murine cell line

Expression vectors	IGF2 gene	Transgene expression in 72 h culture (pg/ml in ELISA assay)	
		mIGF2	hIGF2
p-BLAST	none	35	0
p-mIGF2	murine	740	n.d.
p-hIGF2	human	n.d.	2337

n.d. = not done

Vaccination with DNA carrying the murine IGF2 isoform (mIGF2) did not elicit antibodies, even when combined with Treg depletion. No protection against intravenous challenge with RMS-p53neu5 cells was induced as well (data not shown).

**Fig. 3:**
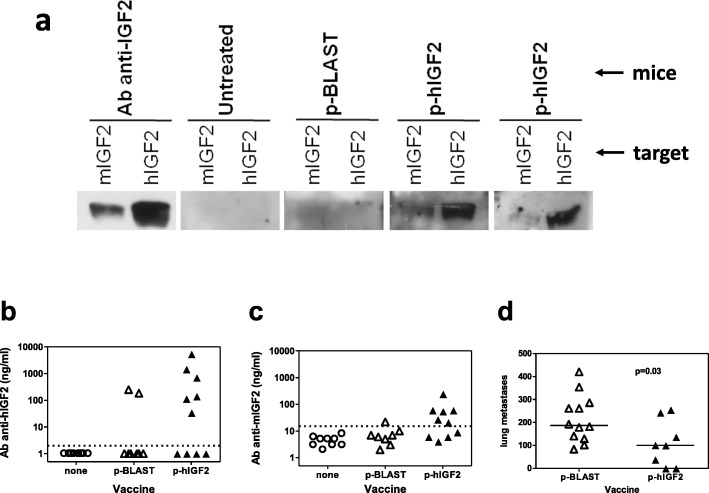
Induction and effectiveness of anti-IGF2 antibodies elicited by electroporated DNA vaccine encoding the human IGF2 gene. **a** Western blot analysis of sera from BALB/c mice untreated or subjected to DNA vaccination and electroporation with empty vector (p-BLAST) or p-hIGF2 (two independent mice are shown). For each mouse, sera were used to stain mIGF2 protein (left lane) or hIGF2 protein (right lane). **b** ELISA assay for anti-hIGF2 antibodies in sera from BALB/c mice untreated (open circles) or vaccinated with empty vector (open triangles) or with p-hIGF2 (closed triangles). Dashed line: sensitivity threshold as determined by the level of untreated mice. **c** ELISA assay for anti-mIGF2 antibodies. Symbols as in panel b. **d** Prevention of RMSp53Neu-5-induced metastasis in mice vaccinated with the empty vector (open triangles, *n* = 12) or with the p-hIGF2 plasmid (closed triangles, *n* = 8) and electroporated. Two similar experiments were pooled. Significance of difference of hIGF2-vaccinated mice versus mock-vaccinated (empty plasmid) was calculated with the non-parametric Wilcoxon test

DNA vaccine for the human IGF2 isoform was able to elicit anti-hIGF2 antibodies which at least partially recognized the murine IGF2 isoform (Fig. 3a). ELISA assay confirmed that the majority of vaccinated mice produced anti-hIGF2 antibodies (Fig. 3b) which also recognized mIGF2 (Fig. 3c). Two mice vaccinated with control p-BLAST vector displayed an over-threshold reactivity against hIGF2, but they were devoid of any reactivity against mIGF2. Mice vaccinated with hIGF2 DNA, producing antibodies cross-reacting with mIGF2, were partially protected from a subsequent injection of RMSp53Neu-5 rhabdomyosarcoma cells, showing a significant 60% decrease in the number of lung metastases when compared with untreated controls (Fig. 3d).

## Discussion

In this paper we investigated the possibility to exploit immunity to interrupt the IGF2-autocrine system involved in the genesis and growth of rhabdomyosarcoma.

Rhabdomyosarcoma is an IGF2-dependent tumor, due to the autocrine overexpression of IGF2 [4]. Both experimental and clinical studies have been performed concerning therapeutic opportunities targeting IGF1R [8, 10]. Since prevention is generally more effective than cure [28–31], we tried to exploit immune approaches to prevent IGF1R-dependent carcinogenic process. As a model of spontaneous rhabdomyosarcoma we used male mice with p53-KO and transgenic for rat HER2 (BALB-p53Neu). These mice develop at early age pelvic rhabdomyosarcomas with IGF2 overexpression, almost concomitantly to IGF2-independent salivary gland carcinomas [19]. The predictable site of onset, a peculiarity of this rhabdomyosarcoma murine model, allowed either the local preventive treatment with neutralizing antibodies and an easy monitoring of tumor onset. In BALB-p53Neu mice carcinogenesis was reported to be partially prevented by anti-HER2/neu cell or DNA vaccines [32, 33].

We showed here for the first time that immune targeting of the autocrine IGF2 delayed IGF1R-dependent rhabdomyosarcoma genesis. Our results confirm the anti-tumor immune targeting of IGFs reported in a few other experimental models, both in preventive and in therapeutic approaches [13–16]. The passive administration of anti-IGFs antibodies was able to delay the onset of rhabdomyosarcoma in BALB-p53Neu mice. Neither off-target effects towards the IGF2-independent salivary carcinoma nor systemic effects on mice growth were observed. Prevention of rhabdomyosarcoma was dose-dependent, confirming our previous studies on cancer immunoprevention showing that a high and prolonged level of antibodies was essential to obtain a maximal preventive effect [34]. Since a high and prolonged level of antibodies is a hard goal to obtain with passive administration, we investigated the possibility to induce in the host itself the production of antibodies neutralizing IGF2.

We used DNA vaccines according to protocols previously found successful against HER2/neu, also combined with adjuvant stimuli, such as a Treg-depleting treatment [24], or the use of xenogeneic antigens [35]. While DNA vaccines easily induced high-level anti-HER2 antibodies, they mostly failed when applied to IGF2. Reasons for this difference could be a leaky tolerance to a transgene (HER2/neu), or, in alternative, the importance of the IGF1R-based system that evolved as a tightly tolerized system [36].

While DNA vaccine for murine IGF2 did not succeed in eliciting antibodies, DNA vaccination with the highly homologous human IGF2 elicited antibodies recognizing murine IGF2. Mice with autochthonously produced anti-IGF2 antibodies were partially protected from an intravenous challenge with IGF2-overexpressing murine rhabdomyosarcoma cells. The use of xenogeneic gene for DNA vaccine has been reported as an adjuvant for tolerance breakage in other systems [35, 37].

## Conclusions

The immune targeting of IGF2 can hamper both the onset and the metastatic growth of IGF1R-addicted rhabdomyosarcoma. IGF2 is a new target which could be neutralized by immune approaches in prevention and therapy of rhabdomyosarcoma.

## Abbreviations

BALB-p53Neu: Mice bearing the p53^+/−^/Neu^+/−^ genotype
IGF1R: Insulin-like Growth Factor receptor-1
IGF2: Insulin-like Growth Factor-2
IGFs: Insulin-like Growth Factors
MAb: Monoclonal Antibody
